# Affordability of antimicrobials for animals and humans in Vietnam: A call to revise pricing policies

**DOI:** 10.1016/j.ijantimicag.2019.05.009

**Published:** 2019-08

**Authors:** Juan Carrique-Mas, Nguyen Van Cuong, Bao Dinh Truong, Doan Hoang Phu, Tran My Phuc, Hugo Turner, Guy Thwaites, Stephen Baker

**Affiliations:** a Oxford University Clinical Research Unit, Ho Chi Minh City, Vietnam; b Centre for Tropical Medicine and Global Health, Oxford University, Oxford, UK

*Sir*,

A recent review in the *International Journal of Antimicrobial Agents* revealed how the practice of purchasing antimicrobials over-the-counter and without a prescription is widespread in most low- and middle-income countries (LMICs) [1].

Antimicrobials for human medicine are commonly acquired without a prescription in Vietnam, despite legislation restricting this practice. Conversely, antimicrobials aimed at veterinary medicine can be legally purchased without prescription by anyone from any of the ∼12 000 veterinary drug stores across the country. It is not known to what extent the ease of access and affordability contributes to excessive antimicrobial usage (AMU) in animal production. Furthermore, there is little information regarding the affordability of antimicrobials in different countries, or how their pricing compares with the equivalent antimicrobial drugs sold in human medicine.

As a component of a study of 270 cycles of production in 112 chicken farms in the Mekong Delta region of Vietnam [2], we identified 236 different products containing 42 different active antimicrobial ingredients. On average, five (interquartile range 2.25–10) antimicrobial products were used per flock cycle, which typically lasts for 16–18 weeks; the majority of AMU occurs during the early (‘brooding’) phase of production.

We calculated the cost incurred for the daily treatment of 1 kg of live chicken (equivalent of administering one animal daily dose kg, ADD_kg_) for the 10 most common antimicrobial products used by farmers. The vast majority of products are powder based, and are administered orally after dilution with water. All products were purchased as 0.1-kg powder sachets. In calculating the dose, we followed the manufacturer's guidelines for their preparation and administration, assuming that a 1-kg chicken typically drinks 225 ml of water per day under local environmental conditions. We expressed the costs in cents of a US dollar (US$) (Table 1).

**Table 1:** The 10 most common antimicrobials used by a cohort of 112 farmers investigated over 270 cycles of production, and the prices of animal daily dose kg (ADD_kg)_

Product	Antimicrobial active principle	Volume (L) of antimicrobial solution prepared per sachet of product (prophylaxis/therapy)	No. of ADD_kg_ per sachet (prophylaxis/therapy)	Cost of 1 ADD_kg_ (range) (in US$ cents)	
				Prophylaxis	Therapeutic
1	Colistin + oxytetracycline	250/100	1111/444	0.07 (0.06–0.17)	0.19 (0.14–0.43)
2	Colistin + oxytetracycline	-/100	-/444	–	0.28 (0.10–0.48)
3	Colistin + gentamicin	-/50	-/222	–	0.44 (0.33–0.62)
4	Colistin + oxytetracycline	100/50	444/222	0.51 (0.29–0.58)	1.02 (0.58–1.16)
5	Oxytetracycline + streptomycin	-/50	-/222	–	0.42 (0.19–0.58)
6	Colistin + oxytetracycline	100/50	444/222	0.20 (0.15–0.43)	0.40 (0.29–0.97)
7	Sulphamethoxazole + thiamphenicol	67/33	296/148	0.51 (0.22–0.72)	1.03 (0.43–1.45)
8	Methenamine	100/67	444/296	0.53 (0.43–0.63)	0.79 (0.65–0.94)
9	Doxycycline + tylosin	400/200	1778/889	0.12 (0.04–0.16)	0.23 (0.07–0.31)
10	Gentamicin + tylosin	100/50	444/222	0.43 (0.14–0.58)	0.85 (0.29–1.15)

NI, not indicated.

Prices are expressed in US$ cents, based on an exchange rate of 1 US$=23 319 VND (23 September 2018)]. The products are sorted by frequency of use. All products were purchased as 100-g sachets.

The price of 1 ADD_kg_ of antimicrobial product ranged from 0.19 to 1.03 US$ cents (average 0.56 US$ cents per kg of chicken). However, in many cases, the product labels include guidelines for prophylactic administration, requiring a lower (≤50%) concentration, and therefore representing less than half of the cost (i.e. on average <0.28 US$ cents per kg of chicken). The most commonly used product contained colistin, which was also the most affordable (0.19 and 0.07 US$ cents for therapeutic and prophylactic use, respectively). As a comparison, the equivalent costs of products containing the same antimicrobial sold for human use in Vietnam per kg recommended therapeutic dose (assuming a 60-kg weight for a human adult) were: thiamphenicol (1.61 US$ cents), gentamicin (0.87 US$ cents), streptomycin (0.78 US$ cents), doxycycline (0.55 US$ cents) and sulfamethoxazole (0.50 US$ cents). Vietnam is among the countries where AMU is expected to increase rapidly in the coming years [3]. It has been suggested that increasing user fees may deter excessive AMU in food animal production, and the increased revenues could be used to mitigate the consequences of antimicrobial resistance [4].

Assuming that a typical chicken is supplemented with antimicrobials for 40 of its 126-day life cycle), with an average weight at treatment of 0.25 kg, this would represent a cost of antimicrobials equivalent to ∼0.03 US$ cents per chicken. Farmers in the Mekong Delta of Vietnam sell their slaughter-age chickens at 6 US$ per bird; thus, the cost of antimicrobials represents approximately 0.5% of the income raised from chicken sales. Although we do not have data on the price of antimicrobials in other animal species or in other LMICs, these prices seem to be remarkably low and are unlikely to be a limiting factor for unnecessary AMU. Directions for use indicating prophylactic dilution contribute to re-inforce the concept that the use of antimicrobials when the flock is healthy is appropriate. More often than not, antimicrobials are sold in combination with vitamins and other health-supporting substances. More worryingly, some of the most commonly used products in animals contain colistin, which is a critically important antimicrobial of the highest priority for human medicine.

Vietnam is an LMIC that does not manufacture active antimicrobial ingredients itself, instead relying on imports. These chemicals are mixed, packed and distributed within the country to meet local demand. We propose that an import tax on antimicrobials of critical importance for human use should be considered. With the exception of ampicillin, amoxicillin and their derivatives (subjected to 5% and 10% import tax, respectively), most antimicrobials are currently exempt of import tax in Vietnam [5]. In order to avoid these increases having a negative impact on the availability of antimicrobials of critical importance for human use when genuinely needed, we recommend effective enforcement of existing legislation to restrict over-the-counter access, while subsidising the use of these antimicrobials if acquired with a doctor's prescription.

An alternative would be to levy a tax for veterinary antimicrobial products. Anecdotal information from our interaction with farmers suggests that the majority would not alter their AMU behaviour substantially, even with a four-fold increase in the price of antimicrobials. Within the proposed tax system, antimicrobials of critical importance used in veterinary medicine should be allocated to the highest tax bracket. There is a risk that such increases may lead to the undesirable creation of a black market of cheap counterfeit products. However, as most farmers are not aware of the differential effectiveness or impact on antimicrobial resistance associated with the use of antimicrobials of critical importance, we believe this would likely result in a preferential choice of ‘older-generation’ types of antimicrobials. The revenues raised from this tax could help train veterinary pharmacists to improve their prescription practices.

There is remarkable diversity in retail prices of antimicrobials for animal use [3] across LMICs, presumably reflecting differences in production capacity, market structure and AMU practices. As such, we propose that such a taxation system should be defined on a country-by-country basis. Crucially, the use of (any) antimicrobials as prophylactic agents should be discouraged in all cases, and veterinary drug manufacturers should make this explicit in the product labels.

## Funding

This work was funded by the Wellcome Trust through an Intermediate Clinical Fellowship awarded to Juan Carrique-Mas (Grant No. 110085/Z/15/Z).

## Competing interests

None declared.

## Ethical approval

Not required.
